# Exploring the Antidiarrheal Properties of Papaya Leaf: Insights *In Vivo* Study in Mice-Model and *In Silico* Analysis at M3 Muscarinic Acetylcholine Receptor Interaction

**DOI:** 10.1155/2024/1558620

**Published:** 2024-06-12

**Authors:** Nyi Mekar Saptarini, Faruk Jayanto Kelutur, Mary Jho-Anne Tolentino Corpuz

**Affiliations:** ^1^Department of Pharmaceutical Analysis and Medicinal Chemistry, Faculty of Pharmacy, Universitas Padjadjaran, Sumedang 45363, Indonesia; ^2^Department of Pharmacy, Faculty of Pharmacy, University of Santo Tomas, Manila 1015, Philippines

## Abstract

Diarrhea caused by gut motility involves 5-HT_3_ serotonin receptor (5-HT_3_R) antagonist, M3 muscarinic acetylcholine receptor (M_3_-AchR), and H_1_ histamine receptor (H_1_R) via their respective agonist. Papaya (*Carica papaya* L.) leaf is an herbal medicine to treat diarrhea in Indonesia, though this has not yet been proven scientifically. This study aimed to determine the antidiarrheal properties of papaya leaf through *in vivo* and *in silico* studies. In the mice model, papaya leaves were infused with distilled water and subjected to *in vivo* antidiarrheal study using castor oil-induced diarrhea. *In silico* molecular docking study of nineteen secondary metabolites was carried out on the M_3_-AchR (PDB ID: 5ZHP) using AutoDock Tools 1.5.6, while ADMET was predicted by pre-ADMET. The results showed that papaya leaf infusion caused a decrease in the total number of feces, an increase in the onset time of diarrhea, a reduction in the frequency of diarrhea, and an increase in the percentage of inhibition of diarrhea. Quercetin 3-rutinoside, a flavonoid glycoside, is potentially an antidiarrheal remedy at M_3_-AchR. ADMET prediction showed good distribution on the target and was not toxic, but absorption needed to be considered. We concluded that the antidiarrheal activity of papaya leaf infusion was dose-dependent. Based on a molecular docking study, the flavonoid glycoside was very effective as an antidiarrheal remedy. ADMET prediction showed a specific distribution to the target and was not toxic.

## 1. Introduction

Diarrheal disease is the third leading cause of child mortality globally, after lower respiratory infections, neonatal preterm birth, neonatal asphyxia, and trauma. Diarrhea is a global problem; almost 1.5 million people died from diarrheal diseases in 2019 [1]. The diarrhea pathogenesis showed that gut motility is related to serotonin (5-hydroxytryptamine) type 3 receptor, 5-HT_3_R [2], muscarinic type 3 acetylcholine receptor (M_3_-AchR) [3], and histamine type 1 receptor (H_1_R) [4]. 5-HT_3_R, M_3_-AchR, and H_1_R have important roles in modulating motility and gastrointestinal (GI) secretion [2–4], where 5-HT_3_R activation is due to increased colonic transit [2]. M_3_-AchR is stimulated by neurotransmitter acetylcholine (ACh), which causes smooth muscle contraction and increased secretion in the GI [3, 5]. Meanwhile, H_1_R regulates food, water intake, and diurnal feeding rhythm [4].

M-AchR, namely, M3, plays an important role in various diseases, such as irritable bowel syndrome (IBS) [5, 6]. Excess ACh within the nervous system controls smooth muscle, cardiac muscle, and endocrine-exocrine functions leading to diarrhea (D-IBS) [5]. The pathophysiology of D-IBS occurs in M_3_-AchR, so it becomes a specific treatment target [3]. Therefore, drugs that block these receptors, such as anticholinergics (loperamide, loratadine, baclofen, amitriptyline, oxybutynin, and chlorphenamine) are needed, which can be used to reduce motility and GI secretion, thereby alleviating the symptoms of diarrhea [7]. However, these drugs have side effects, such as headaches, memory problems, decreased cognitive function, behavioral disorders, anxiety, and insomnia [8].

The World Health Organization (WHO) supports efforts to improve the safety and efficacy of herbal medicines. In addition, it is recommended that herbal medicine is used in maintaining public health and preventing and treating diseases because of the fewer side effects. About 80% of the world's population in developing countries, including Indonesia, use herbal medicine for their primary health [9]. So far, only guava (*Psidium guajava* L.) leaves are available in the Indonesian market. So, it needs to be developed using other herbal ingredient medicines with the same potential as antidiarrhea, such as papaya (*C*. *papaya* L.) leaves.

Papaya, a flowering plant in the *Caricaceae* family, is native to tropical America, i.e., Southern Mexico and neighboring Central America. It has spread to tropical countries, including Indonesia [10, 11]. In Indonesia, the area and production of papaya reached 107,813 hectares, with a total production of 1,089,578 tons in 2022 [12]. Papaya leaves have been used for lactation, medicine for cancer, digestion, and dengue fever [13]. It also has an activity of anti-inflammatory, antidiabetic, immunomodulatory, antiviral, increased platelet [14], antimalarial, and antiplasmodial [15]. Papaya leaves showed no harmful effects on long-term administration [16]. Phytochemical screening showed that papaya leaves contain saponins, cardiac glycosides, anthraquinones, reducing sugars, flavonoids, alkaloids, and tannins [17]. Even though papaya has several critical medicinal properties, studies have yet to be performed to investigate its antidiarrheal properties. Therefore, this study used an experimental (animal model) approach to examine the antidiarrheal activity of secondary metabolites in the infusion of papaya leaves and a computational *in silico* molecular docking and ADMET prediction for the first time.

## 2. Materials and Methods

### 2.1. Medicine and Chemicals

Loperamide HCl, PGA (Pulvis Gummi Arabicum or acacia gum), and castor oil were pharmaceutical grade and purchased from Hebei Yingong New Material Technology Co., Ltd. (China). All chemicals in phytochemical screening were analytical grade and purchased from Merck (Germany).

### 2.2. Plant Collection and Identification

47.38 g of papaya leaves produced 15.44 g of dried leaves. The leaves were obtained from the Manoko plantation, Lembang district, West Java, Indonesia, in July 2021. The plant was identified in the Plant Taxonomy Laboratory, Faculty of Mathematics and Natural Sciences, Universitas Padjadjaran, Indonesia, with No. 273/HB/07/2021.

### 2.3. Experimental Animals

Swiss Webster male mice aged 2-3 months with 20–25 g of body weight and in a healthy condition were used as experimental animals. Mice were sheltered in polypropylene cages by maintaining laboratory conditions (room temperature 25 ± 2°C, relative humidity 65–70%; 12 h light/dark cycle) and standard laboratory food and distilled water ad libitum. The animals were acclimatized to laboratory conditions for 7 days before the experiment.

### 2.4. Ethical Clearance

*In vivo* experimental procedures and protocols in this study were reviewed and approved by the Health Research Ethics Committee, Faculty of Medicine, Universitas Padjadjaran with No. 2142/UN6.KEP/EC/2021.

### 2.5. Infusion Preparation and Phytochemical Screening

4, 8, and 16 g of papaya leaves were extracted with 100 mL of distilled water for 15 min at 90°C. Infusion was filtered to 100 mL of volumetric flask and diluted to volume with distilled water [18]. The infusion concentration was 40, 80, and 160 mg/mL, respectively. Phytochemical screening was conducted with methods described by Tiwari et al. [19] and Benne et al. [20] to identify polyphenols, flavonoids, tannins, alkaloids, saponins, monoterpenoids, terpenoids, and steroids.

The infusion reacted with (a) sodium hydroxide solution will develop a dark yellow solution, then become colorless if diluted acid is added, and (b) lead acetate solution will develop yellow precipitate. The color change indicates flavonoids presence. The infusion is treated with ferric chloride solution and will develop a bluish-black solution to indicate phenols' presence. The infusion added to a 1% gelatin solution containing sodium chloride will develop a white precipitate to indicate tannins' presence. The infusion is shaken for 15 min to develop (a) a foam layer 1 cm thick and (b) a foam lasting for 10 min. The foams indicate saponins' presence [19, 20].

The infusion is dissolved in dilute hydrochloric acid and filtered. (a) The filtrate is reacted with Mayer's reagent to develop a yellow precipitate. (b) The filtrate is made to react with Wagner's reagent to develop a brown or reddish precipitate. (c) The filtrate is reacted with Dragendroff's reagent to develop a red precipitate. The various precipitates indicate alkaloids' presence [19, 20].

The infusion is evaporated until dry, and then extracted with chloroform. We add 5 drops of acetic anhydride followed by sulfuric acid from the side of the test tube to develop a blue ring at the junction of the two fluids indicating steroids' presence. The infusion added to 2 mL of chloroform, shaken, and filtered. We add 5 drops of concentrated sulfuric acid to the filtrate and shake to develop a yellow precipitate, which indicate triterpenoids' presence [20].

### 2.6. Antidiarrheal Activity Assay against Castor Oil-Induced Diarrhea in Mice

Mice fasted for 24 hours and were randomly divided into five groups of five mice in each group (*n* = 5). Group I was administered 0.5 mL of 1% PGA suspension orally and served as a negative control (NC). Group II was administered 0.5 mL of loperamide HCl (5.2 *μ*g/mL) orally and served as a positive control (PC). Groups III, IV, and V were administered an oral dose of 0.5 mL of papaya leaf infusion at 40, 80, and 160 mg/mL, respectively. One hour after administering the test doses, the mice received 0.5 mL castor oil orally to induce diarrhea, then individually placed in cages with the floor lined with filter paper to observe the total number of feces, onset time of diarrhea, and frequency. The total feces were weighed every 30 min for 3 hours, and the papers were changed every hour after each evaluation. The mean number of control group feces was considered to be 100%. The percentage inhibition of diarrhea (%) for all the groups was calculated compared to the negative control by using the following equation: inhibition (%) = ((TD control – TD test group)/TD control) × 100, where TD control = total feces of the negative control group and TD test group = total feces of the test or positive control group [21].

### 2.7. Selection of Compounds for Molecular Docking Study

The native ligand was separated from water molecules and protein using Discovery Studio 2016 Client® (DS) (BIOVIA, San Diego, CA, USA) [22] by the script menu, and the selected water molecules and protein chains were saved [23]. The alkaloids include carpaine, dehydrocarpaine I, dehydrocarpaine II, and emetine. Flavonoids and flavonoid glycosides include quercetin 3-(2-rhamnosylrutinoside), kaempferol 3-(2-rhamnosylrutinoside), quercetin 3-rutinoside, myricetin 3-rhamnoside, quercetin, and kaempferol. The phenol group and phenolic acid include chlorogenic acid, ferulic acid, gallic acid, 5,7-dimethoxycoumarin, caffeic acid, o-coumaric acid, p-coumaric acid, protocatechuic acid, and (*E*)-3-(hydroxy-3-(3,4,5-trimethoxy benzyl) phenyl) acrylic acid as well as loperamide as a comparison (drug). These compounds were selected based on a characterized chemical structure [24]. The chemical structures were obtained from the PubChem database (https://pubchem.ncbi.nlm.nih.gov/) in a 2D structure SDF format. Meanwhile, ligands use AutoDock Tools 1.5.6 (ADT) (The Scripps Research Institute, USA) [25] by adding compute Gasteiger, all hydrogen, and merge non-polar [26].

### 2.8. Molecular Docking and ADMET Prediction for Antidiarrheal Activity

The three-dimensional X-ray crystal structure of the protein used for molecular docking was obtained from the Protein Data Bank (https://www.rcsb.org/), i.e., M_3_-AchR (PDB ID: 5ZHP). Molecular docking was conducted with DS and ADT. Protein was separated from native ligands using DS [22]. Then, it was prepared again with ADT by adding the Kollman charge and hydrogen polar [23]. Parameter setting of the grid and docking was carried out in ADT. Determination of the grid box on the active site in the form of the box parameter location and the grid box size (distance, Å) using the ADT [27]. The result obtained was the coordinate of the central grid point (*x*, *y*, and *z*) used as a reference for docking papaya leaf's phytochemical constituents to the M_3_-AchR. For docking parameters based on the Lamarckian genetic algorithm (100 times) with a value of 27,000 (algorithm generation), 2,500,000 (energy evaluation), as well as 150 (population) [28].

The software parameter was validated with the root mean standard deviation (RMSD) value of the result of redocking as close as the crystallographic value in the selected active site area [29]. The molecular docking process for the compounds of papaya leaves was carried out the same way as the validation process, which uses grid parameters and the docking of the validation result. The results were observed in the form of binding affinity, including free energy of binding (Δ*G*) and inhibition constant (Ki), as well as amino acid residues [30]. The output of molecular docking was a notepad file, and then the lowest bond energy value (best pose) was selected based on the cluster rank. Visualization of the position and orientation of compounds when interacting with protein, the amino acids, and bonds formed using the DS [22]. At the same time, pharmacokinetic properties from constituents of papaya leaves were predicted by pre-ADMET (https://preadmet.bmdrc.kr/).

### 2.9. Statistical Analysis

Results were presented as mean ± standard error of the mean. All comparisons were made by using one-way ANOVA followed by Dunnett's test. A *p* value of less than 0.05 was considered statistically significant.

## 3. Results

### 3.1. Infusion Preparation and Phytochemical Screening

A total of 47.38 g of papaya leaves produced 15.44 g of dried leaves, so the yield was 32.59%. The percentage yield shows that the papaya leaves had less water content than others. The infusion of papaya leaves was light green with a characteristic papaya odor. Phytochemical screening based on color change or precipitation showed that papaya leaves contain polyphenols, tannins, flavonoids, alkaloids, and saponins.

### 3.2. Antidiarrheal Activity Assay against Castor Oil-Induced Diarrhea in Mice Model

**Figure 1:**
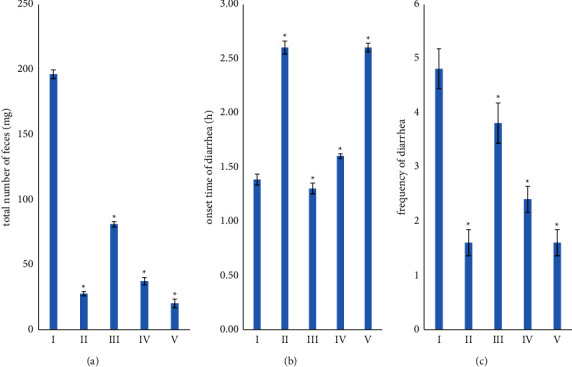
The result was in five different groups (a) total number of feces (mg), (b) onset time of diarrhea (h), and (c) frequency of diarrhea. Group I was negative control, group II was loperamide HCl, group III was infusion 40 mg/mL, group IV was 80 mg/mL, and group V was infusion 160 mg/mL. ^*∗*^Significantly different when compared with the negative control group at *p* value <0.05, and values were expressed as mean ± SEM (*n* = 5).

The results of castor oil-induced diarrhea are shown in Figure 1. There were significant differences in the total number of feces (*p* value = 2.19 × 10^–21^), the onset time of diarrhea (*p* value = 1.10 × 10^−11^), and the frequency of diarrhea (*p* value = 4.62 × 10^−7^). The percentage of inhibition of diarrhea from loperamide HCl was 85.93%, while from infusion 40, 80, and 160 mg/mL were 58.72, 81.04, and 89.70%, respectively.

### 3.3. Molecular Docking Study for Antidiarrheal Activity

**Figure 2:**
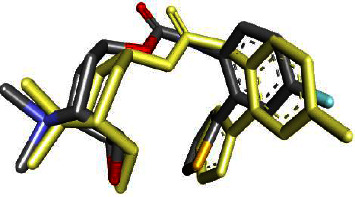
Overlap native ligand of the crystallographic value (yellow) with redocking (grey).

The validation method for the software was declared valid and is presented in Table 1. This is because the redocking ligand value (0.996 Å) was smaller than the crystallographic value (3.10 Å). Visualization of native ligand cocrystal by redocking is shown in Figure 2.

Table 2 of the molecular docking results shows that the flavonoid from papaya leaves had a good docking score compared to other compounds. The docking results of triplicates can be seen in the supplementary data (available here). It is based on the binding affinity value, where quercetin 3-rutinoside was −11.50 kcal/mol and 3.75 nM against the M_3_-AchR. Although quercetin 3-rutinoside, a flavonoid containing glycosides, has a binding affinity value that is slightly different from the standard drug, it is not so significant that it has potential as an antidiarrheal remedy. The group of alkaloids (carpaine and dehydrocarpaine II) has no interaction with M3-AchR because of the value of Δ*G* > 0. It showed that not all alkaloid secondary metabolites from papaya leaves have a binding affinity to the active sites of the receptor [31].

**Figure 3:**
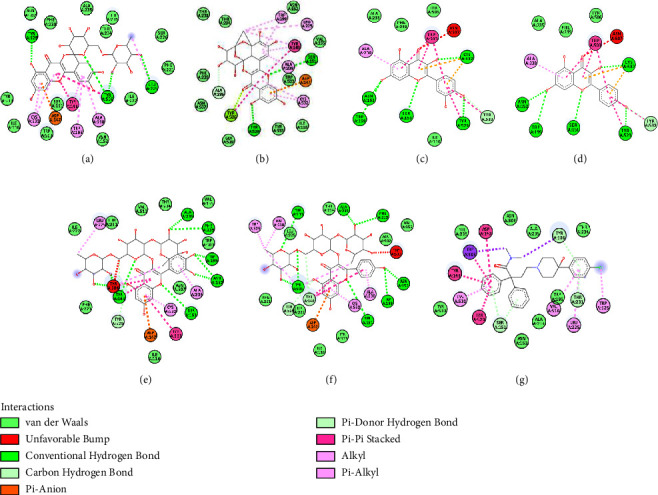
The 2D interactions of the best pose for (a) quercetin 3-rutinoside, (b) myricetin 3-rhamnoside, (c) quercetin, (d) kaempferol, (e) quercetin 3-(2-rhamnosylrutinoside), (f) kaempferol 3-(2-rhamnosylrutinoside), and (g) loperamide when docked to the M_3_-AchR.

**Figure 4:**
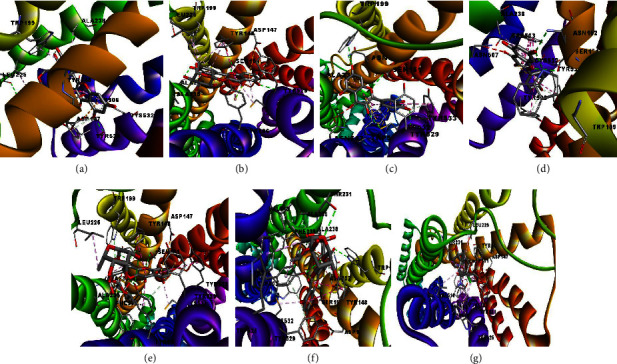
3D interactions based on the best-ranked pose of (a) quercetin 3-rutinoside, (b) myricetin 3-rhamnoside, (c) quercetin, (d) kaempferol, (e) quercetin 3-(2-rhamnosylrutinoside), (f) kaempferol 3-(2-rhamnosylrutinoside), and (g) loperamide in the binding pocket of M_3_-AchR.

A study of the docking fits of each compound has different interactions when it binds to the target protein or enzyme. Table 2 shows the hydrogen bonds between compounds with M_3_-AchR. The compounds formed at least three hydrogen bonds (conventional, carbon, and pi-donor hydrogen bonds) with various amino acid residues, except for the alkaloid group, which tends to form Van der Waals bonds. Molecular docking for flavonoids and flavonoid glucosides is shown in Figure 3 for 2D and Figure 4 for 3D.

Pharmacokinetic properties include absorption (human intestinal absorption (HIA) and human colon adenocarcinoma cells (Caco-2)), distribution (plasma protein binding (PPB) and blood-brain barrier (BBB)), metabolism (CYP2D6 and CYP3A4 as substrate and inhibition), and toxicity test (mutagenic and carcinogenic) were predicted in compounds of papaya leaves before further stages (clinical trials).  Table 3 shows that kaempferol and loperamide have an absorption value between 70–100%, which showed promising results when in the intestine. At the same time, the other compounds were moderately and poorly absorbed.

Caco-2 showed medium cell permeability because the value was 4–70 nm·sec^−1^ in all constituents from papaya leaves. The PPB value for all compounds was less than 90%, meaning the plasma protein binding was weak, except for loperamide (96.8%). Predicted results of BBB (log BB) on loperamide (11.08) can easily cross membrane permeability because log BB >0.3. Metabolism predictions show that quercetin 3-rutinoside, myricetin 3-rhamnoside, quercetin, kaempferol, quercetin 3-(2-rhamnosylrutinoside), and kaempferol 3-(2-rhamnosylrutinoside) are neither substrates nor inhibitors of CYP2D6, except loperamide. Meanwhile, quercetin 3-rutinoside, myricetin 3-rhamnoside, quercetin 3-(2-rhamnosylrutinoside), kaempferol 3-(2-rhamnosylrutinoside), and loperamide were weak substrates for CYP3A6. Toxicity shows that quercetin 3-(2-rhamnosylrutinoside) and kaempferol 3-(2-rhamnosylrutinoside) were mutagen and carcinogen, except quercetin 3-rutinoside and myricetin 3-rhamnoside which were neither. Only quercetin 3-rutinoside, myricetin 3-rhamnoside, quercetin, kaempferol, and loperamide are not carcinogenic.

## 4. Discussion

Almost all parts of *C*. *papaya* are used as medicine, such as the fruit as an antidiarrheal remedy [32] and the leaves have activity as antioxidant, antihypertensive, wound-healing, hepatoprotective, anti-inflammatory, antimicrobial, antifungal, antifertility, antihistaminic, antidiuretic, antiamoebic, antitumor, anthelmintic, antimalarial, antihyperglycemic, immunomodulatory, antiulcer, and antisickling [24]. The study conducted by Unaeze et al., 2018, reported that papaya leaf extract has a minimum inhibitory concentration and minimum bactericidal concentration of 25 to 50 mg/mL and 50 to 100 mg/mL, respectively. These results show that papaya leaves are recommended as an antidiarrheal remedy [33].

The infusion was chosen as the extraction method in this study because it adjusts the empirical use of papaya leaves in Indonesian society, which are boiled for 15 min using water. It causes a high concentration for the *in vivo* test. The results of the phytochemical screening of papaya leaves from Bandung were the same as those of Patil et al. [17]. Therefore, the secondary metabolites for molecular docking and ADMET prediction were taken from the secondary metabolites that have been characterized. Papaya leaves contain various phenolic compounds, such as kaempferol, protocatechuic acid, quercetin, 5,7-dimethoxy coumarin, caffeic acid, *p*-coumarin acid, and chlorogenic acid. These compounds are also the major isolates in the leaves, i.e., quercetin 3-O-*α*-1C4-rhamnopyranoside, quercetin-3-O-glucopyranoside, and quercetin-3-O-rutinoside [34, 35].

Castor oil contains ricinoleic acid, which irritates the intestinal mucosa, causing inflammation and release of prostaglandins, stimulating gastrointestinal secretion, motility, epithelial permeability, and edema of the intestinal mucosa. This prevents sodium, chloride, and water reabsorption, thus causing diarrhea [36]. Loperamide HCl is an antidiarrheal standard due to antagonist diarrhea induced by castor oil due to antisecretory and antimotility activities. It blocks M_3_-AchR, causing muscle relaxation [37]. Papaya leaf infusion exhibited significant antidiarrheal activity in suppressing the propulsive movement of gastrointestinal transit, which indicates the capability to reduce the frequency of feces in diarrhea conditions [38]. The same study was conducted by Terefe et al., 2023, on animals screened to respond to castor oil by inducing watery diarrhea with an effective 400 mg/kg dose. It is because castor oil induces diarrhea by releasing nitrate oxidation, thereby increasing the permeability of the GI membrane to calcium, stimulating prostaglandin synthesis, and causing increased fluid and electrolyte entry into the bowel lumen and peristalsis [39].

Acetylcholine acts on M3 muscarinic receptors to initiate intestinal motility [40]. Antidiarrheal activity is predicted to be caused by the relaxant activity of antagonizing muscarinic acetylcholine receptors. Medicinal plants containing secondary metabolites with complementary pharmacology activity and synergism mechanisms are predicted to treat various diseases [41]. Several parts of plants, extracts, and plant-derived products have been widely used as medicine [42]. Plants are generally rich in secondary metabolites with various biological activities, which naturally act as a defensive mechanism against bacteria, insects, viruses, and fungi [41].

Metabolites of alkaloids, flavonoids, phenols, tannins, terpenoids, and saponins have antibacterial and anthelmintic activities [43, 44]. In addition, various extracts from plants show antidiarrheal effects due to reactions from saponins, tannins, steroids, flavonoids, and alkaloids. Meanwhile, tannins and flavonoids can aid reabsorption of intestinal fluids and electrolytes [45]. Tannins can also reduce intestinal motility by inhibiting bowel irritation [46].

The *in vivo* results showed a significant difference between the negative control, test, and positive groups, i.e., loperamide HCl. Figure 1 shows that the increased infusion dose was proportional to the increased antidiarrheal activity. These were observed from a decrease in the total number of feces, an increase in the onset time of diarrhea, a reduction in the frequency of diarrhea, and an increase in the percentage of inhibition of diarrhea. It concluded that the antidiarrheal activity of papaya leaf infusion was dose-dependent.

The aqueous papaya leaves extract good antidiarrheal activity and are safe at 200 mg/kg in the rats' model [32]. This study showed the best antidiarrheal activity by infusion of 4000 mg/kg, equal to loperamide HCl as the standard. The infusion dose was observed to be 20 times higher than the extract dose. However, if converted from the yield of 8.78% of the extract, 200 mg of the extract would be equivalent to 2.28 g of leaves [32], while 4000 mg infusion was equivalent to 16 g of leaves. So, the infusion dose was only 7.01 times higher than the extract. It was due to a different extraction method, i.e., maceration was conducted for 3 days using methanol [32], while infusion was performed for 15 min using distilled water. The dose difference showed the different concentrations of the extracted secondary metabolites. Applying papaya leaf infusion has two advantages, i.e., the content of secondary metabolites that have antidiarrheal activity and water to prevent dehydration. This finding may support the acclaimed effect of papaya leaves as an antidiarrheal agent in traditional remedies to develop into herbal medicine. Herbal medicine has a more significant role in developing countries in overcoming drug resistance, toxicity, adverse effects, and increasing costs if synthetic products are used [47].

Papaya fruit has been proven to have antidiarrheal activity [32]. Our study is the first to determine papaya leaf's antidiarrheal activity in a mice model induced by castor oil. This study was conducted based on the results of our phytochemical screening, which showed the presence of polyphenols, tannins, flavonoids, alkaloids, and saponins in papaya leaves. The secondary metabolite content of papaya leaf is similar to papaya fruit, namely, carbohydrates, tannins, saponins, proteins and amino acids, alkaloids, phenolic compounds, and phytosterols [32]. We chose papaya leaves over fruit because papaya leaves are always available, while papaya fruit is available, and you have to wait to flower until it finally produces fruit. This is the novelty and advantage of our study if the papaya leaf is developed into a candidate for herbal antidiarrheal preparations because around 80% of the world's people, including Indonesian, have used herbal plants as a substitute for chemical drugs, mainly for reasons of side effects and expenses [48]. Antidiarrheal activity of medicinal plants is caused by tannins, flavonoids, alkaloids, saponins, reducing sugars, sterols, and glycosides [49, 50]. The antidiarrheal activity of papaya leaf infusion is attributed to the presence of secondary metabolites. Papaya leaf infusion contains polyphenols, tannins, flavonoids, alkaloids, and saponins. Flavonoids inhibit the release of autacoids and prostaglandins [51, 52]. Tannins make intestinal mucosa more resistant, reduce secretion, and stimulate normalization of water transport across the mucosal cells, and reduce intestinal transit [53]. Saponins inhibit histamine release [52]. Flavonoids, tannins, and phenolic compounds also form complex cell walls, binding to adhesion, thus providing antimicrobial activity [50, 53]. These provide a scientific finding for the potential of papaya leaves in antidiarrhea. Papaya leaf infusion suppressed the movement of gastrointestinal transit, which is shown from the onset time and frequency of feces in diarrheal conditions.

Computational studies were effectively used to predict the binding affinity of target ligands and very well demonstrated possible molecular mechanisms for pharmacological response [54]. Binding affinity is an important factor that needs to be considered when ligand-receptor interactions occur. If the binding affinity is low, the ligand (compound) requires less energy to bind to the receptor. So, low binding affinity values have more significant potential to interact with target macromolecules [55]. Free energy of binding analysis aims to determine the spontaneity of a reaction and the stability of the interaction between the ligand receptors indicated by negative Δ*G*. In addition, the stability of the interaction is proportional to the compound's potential when it forms strong chemical bonds [56]. The smaller the number or the negative of Δ*G* indicates a lot of energy to form a stronger bond [57]. Experimentally, Δ*G* was closely related to Ki. According to the equation of Δ*G* = −RT ln Ki, the value of Δ*G* was used to predict the ability of compounds to inhibit macromolecules [58]. In the present study, nineteen secondary metabolites in three major compounds from the papaya leaves, i.e., alkaloids, flavonoids and flavonoid glycosides, as well as phenol and phenolic acid, were investigated against M_3_-AchR and the docking scores obtained for all compounds have been reported in Table 2.

The amino acids Ser151 and Tyr506, which form hydrogen bonds when the ligand binds to M_3_-AchR, play an important role in antidiarrheal activity [5, 6]. Figures 2 and 3 show that quercetin 3-rutinoside has the amino acid residue Tyr506 when forming conventional hydrogen bonds. For myricetin 3-rhamnoside, quercetin, kaempferol, and quercetin 3-(2-rhamnosylrutinoside) form a conventional hydrogen bond with amino acid Ser151. Meanwhile, kaempferol 3-(2-rhamnosylrutinoside) and loperamide form amino acid residues Ser151 and Tyr506 with conventional, carbon, and phi-donor hydrogen bonds.

The development of a drug substance needs to pay attention to its pharmacokinetic properties, as shown in Table 3. HIA shows the percentage of drug absorbed from the cumulative excretion ratio in urine, bile, and feces. Caco-2 is widely used as a model in *in vitro* testing when predicting human drug absorption through the intestinal epithelial cell barrier [59]. The PPB parameter is related to the ability of the drug disposition to exert an effect [60]. At the same time, BBB is used to characterize the distribution of compounds to the blood-brain barrier membrane permeability [59]. Cytochrome P450 (CYP450) is an important enzyme system for drug metabolism in the liver, of which CYP2D6 and CYP3A4 are among its subtypes [61]. Among CYP enzymes, CYP3A4 and CYP2D6 are the most relevant since they metabolize about 50% and 30% of the drugs on the market, respectively [62]. Toxicity in the form of mutagenic and carcinogenic determines the characteristics of compounds causing cell or organ damage when in the body [63].

Quercetin 3-rutinoside has potential antidiarrheal activities based on studies *in silico* molecular docking. The presence of quercetin 3-rutinoside in papaya leaves has been proven using ultra performance liquid chromatography-time of flight-electrospray ionization-mass spectrometry methods, together with six other flavonoids, namely, quercetin, quercetin 3-(2G-rhamnosylrutinoside), kaempferol, kaempferol 3-rutinoside, kaempferol 3-(2G-rhamnosylrutinoside), and myricetin 3-rhamnoside [64, 65]. This result of molecular docking was also supported by reports on the research results that have been performed on bayberry fruit [61]. However, ADMET predictions suggest poor absorption in the gastrointestinal tract and medium permeability in Caco-2 cells. Meanwhile, the distribution has a weak bound so that it can diffuse out of the circulatory system and work specifically on the treatment target [63], and the log BB was not more than 0.3, so it is possible that it is not easy in the blood-brain barrier [66]. Prediction of CYP450 showed neither substrate nor inhibitor to CYP2D6, while CYP3A4 inhibitor showed the substrate was weak. The toxicity test showed that it was neither mutagenic nor carcinogenic; thus, it is safe in the body.

## 5. Conclusions

The phytochemical screening of papaya leaves obtained polyphenols, tannins, flavonoids, alkaloids, and saponins. The antidiarrheal activity of papaya leaves infusion was dose-dependent, which the best infusion being 4000 mg/kg. Quercetin 3-rutinoside, a flavonoid glycoside, with −11.50 kcal/mol and 3.75 nM is potentially an antidiarrheal remedy at M_3_-AchR compared as the target to other compounds. However, the prediction of pharmacokinetic properties needs to be considered on several parameters. ADMET prediction showed a specific distribution to the target and was not toxic, but absorption needed to be considered.

## Figures and Tables

**Table 1:** Result of the validation method.

Docking coordinate value			Grid box size	Grid spacing (Å)	Lamarckian genetic algorithm	RMSD value (Å)	
X	Y	Z				Cocrystal	Redocking
−22.739	−48.565	196.175	40 × 40 × 40	0.375	100	3.10	0.996

**Table 2:** Docking result of phytochemical constituents of papaya leaves to M_3_-AchR.

Compounds	Amino acid residue interaction		Δ*G*^*∗*^ (kcal/mol)	Ki^*∗*^ (nM)
	Hydrogen bond	Van der Waals bond		
*Alkaloids*				
Carpaine	Ser151	Phe221, Ile222, Pro228, Thr234, Trp199, Ala235, Ala238, Phe239, Asn507, Val155, Leu225, and Tyr148	+22.74	—
Dehydrocarpaine I	—	Ser151, Trp503, Val155, Asn152, Thr234, Ala235, Val510, Thr231, Trp525, and Tyr529	−5.34	122747
Dehydrocarpaine II	Ser151	Trp525, Ile222, Val510, Thr234, Ala235, Trp199, Ala238, Asn152, and Val155	+10.67	—
Emetine	Ser151, Asn507, Asn152, Ala238, Trp503, Asp147	Tyr533, Ile116, Tyr529, Thr231, Val510, Thr234, and Val155	−7.19	5363

*Flavonoids and flavonoid glycosides*				
Quercetin 3-(2-rhamnosylrutinoside)	Ala235, Phe239, Trp199, Asn152, Ser151, Tyr148, Tyr529, and Tyr506	Ile116, Phe221, Ile222, Thr231, Val510, Thr234, Val155, and Asn507	−8.14	1166
Kaempferol 3-(2-rhamnosylrutinoside)	Thr231, Ala235, Phe239, Asn152, Trp199, Ser151, Tyr506, Tyr148, and Tyr529	Ile116, Tyr533, Ile222, Phe221, Leu225, Thr234, Asn507, and Val155	−7.86	1753
Quercetin 3-rutinoside	Tyr529, Tyr506, and Leu225	Asn152, Ser151, Trp503, Ile116, Tyr533, Asn507, Phe239, Ala235, Thr231, Thr234, Ser226, Phe221, and Ile222	−11.50	3.75
Myricetin 3-rhamnoside	Ser151, Tyr529, and Ala235	Gly528, Asn507, Phe239, Thr234, Thr231, Asn152, Val155, Trp503, Ile116, and Tyr533	−10.74	13.52
Quercetin	Trp199, Asn152, Ser151, Tyr529, Cys532, and Tyr533	Ile116, Ala235, Phe239, and Tyr506	−8.66	450.07
Kaempferol	Asn152, Trp199, Ser151, Tyr529, Tyr533, and Cys532	Ala235, Phe239, and Tyr506	−8.57	519.89

*Phenol and phenolic acid*				
Chlorogenic acid	Ser151, Tyr529, Tyr506, and Tyr533	Asn152, Ala238, Thr234, Trp199, Ala235, Phe239, Trp503, Asn507, and Asp147	−8.53	540.26
Ferulic acid	Asn507, Cys532, Trp503, Asn152, and Trp199	Phe239, Ala238, Ser151, Tyr148, and Tyr529	−5.50	93820
Gallic acid	Tyr148, Trp199, Asn152, and Ser151	Val155, Phe239, Trp503, and Tyr529	−4.62	411873
5,7-Dimethoxycoumarin	Asn152, Cys532, and Asp147	Trp199, Tyr148, Ser151, Tyr529, Tyr506, Asn507, and Trp503	−6.56	15543
Caffeic acid	Cys532, Asn507, Asn152, and Trp199	Phe239, Ser151, Trp503, and Ala238	−5.58	81443
o-Coumaric acid	Ser151, Asn507, and Tyr148	Asp147, Trp503, Phe239, and Tyr506	−5.75	61403
*p*-Coumaric acid	Cys532, Asn507, Trp199, and Asn152	Tyr148, Phe239, and Ala238	−5.20	155090
Protocatechuic acid	Trp199, Asn152, Tyr148, and Ser151	Trp503, Val155, Tyr506, and Tyr529	−4.56	455450
(*E*)-3-(Hydroxy-3-(3,4,5-trimethoxybenzyl) phenyl) acrylic acid	Ile222, Ser151, Tyr148, Tyr529, and Asp147	Phe221, Cys532, Thr234, Trp199, Ala238, and Asn152	−8.30	828.12

*Comparison (drug)*				
Loperamide	Tyr506, Thr231, Ser151, Ile222, Thr231, Trp525, Tyr148, and Cys532	Tyr533, Phe239, Asn507, Ala235, Thr234, Trp199, Ala238, and Asn152	−11.61	3.10

^*∗*^Δ*G* and KI in Table 2 is the average value of three repetitions.

**Table 3:** ADMET prediction from the compounds of papaya leaves.

Compounds	Absorption		Distribution		Metabolism		Toxicity	
	HIA (%)	Caco-2 (nm sec^−1^)	PPB (%)	BBB (log BB)	Substrate	Inhibition	M^*∗*^	C^*∗*^
					CYP2D6	CYP2D6		
					CYP3A4	CYP3A4		
					Substrate	Inhibitor		
Quercetin 3-rutinoside	2.9	7.9	43.9	0.03	Non	Non	−	−
					Weakly	Inhibitor		
Myricetin 3-rhamnoside	11.7	6.1	65.4	0.03	Non	Non	−	−
					Weakly	Inhibitor		
Quercetin	63.5	3.4	93.2	0.17	Non	Non	+	−
					Non	Inhibitor		
Kaempferol	79.4	9.6	89.6	0.29	Non	Non	+	−
					Non	Inhibitor		
Quercetin 3-(2-rhamnosylrutinoside)	0.6	9.1	35.3	0.03	Non	Non	+	+
					Weakly	Inhibitor		
Kaempferol 3-(2-rhamnosylrutinoside)	1.5	10.0	35.2	0.03	Non	Non	+	+
					Weakly	Inhibitor		
Loperamide	94.9	47.5	96.8	11.08	Substrate	Inhibitor	+	−
					Weakly	Inhibitor		

M = mutagenic and C = carcinogenic.

## Data Availability

The data used to support the findings of this study are available from the corresponding author on request. Docking results of triplicates can be seen in the supplementary data.

## Supplementary Materials

Supplementary Materials Docking results of triplicates of phytochemical constituents of papaya leaves to M3-AchR.

## References

[1] Ourworldindata (2019). Causes of death in children under five.

[2] Crowell M. D. (2004). Role of serotonin in the pathophysiology of the irritable bowel syndrome. *British Journal of Pharmacology*.

[3] Grover M., Camilleri M. (2014). Ramosetron in irritable bowel syndrome with diarrhea: new hope or the same old story?. *Clinical Gastroenterology and Hepatology*.

[4] Fabisiak A., Włodarczyk J., Fabisiak N., Storr M., Fichna J. (2017). Targeting histamine receptors in irritable bowel syndrome: a critical appraisal. *Journal of Neurogastroenterology and Motility*.

[5] Ghorpade R., Kumar D., Nayak S., Acharya B. N. (2019). Design and synthesis of muscarinic acetylcholine receptor (mAChR) antagonist: pharmacophore-based screening and structure-based optimization. *Monatshefte Für Chemie-Chemical Monthly*.

[6] Liu H., Hofmann J., Fish I I. (2018). Structure-guided development of selective M3 muscarinic acetylcholine receptor antagonists. *Proceedings of the National Academy of Sciences*.

[7] McCartney M. (2015). Drugs with anticholinergic side effects and cognitive decline-cause or effect?. *BMJ*.

[8] Ghossein N., Kang M., Lakhkar A. D. (2022). Anticholinergic medications.

[9] Azaizeh H., Fulder S., Khalil K., Said O. (2003). Ethnobotanical knowledge of local Arab practitioners in the Middle Eastern region. *Fitoterapia*.

[10] Saeed F., Arshad M. U., Pasha I. (2014). Nutritional and phytotherapeutic potential of papaya (*Carica papaya* Linn.): an overview. *International Journal of Food Properties*.

[11] Vij T., Prashar Y. (2015). A review on medicinal properties of *Carica papaya* L. *Asian Pacific Journal of Tropical Disease*.

[12] Bps (2020). Production of fruit plants.

[13] Otsuki N., Dang N. H., Kumagai E. (2010). Aqueous extract of Carica papaya leaves exhibits anti-tumor activity and immunomodulatory effects. *Journal of Ethnopharmacology*.

[14] Sudhakar N., Vidhya R. M. T. (2014). Potential medicinal properties of Carica papaya Linn – a mini-review. *International Journal of Pharmacy and Pharmaceutical Sciences*.

[15] Yogiraj V., Goyal P. K., Chauhan C. S., Goyal A., Vyas B. (2014). *Carica papaya* linn: an overview. *International Journal of Herbal Medicine*.

[16] Vaishali M., Shukla R. N. (2014). Comparative study of *Carica papaya* Linn. leaves with synthetic drug diclofenac sodium for its anti-inflammatory activity. *International Journal of Current Research in Chemistry and Pharmaceutical Sciences*.

[17] Patil T., Patil S., Patil A A., Patil S. (2014). *Carica papaya* leaf extract–An ethnomedicinal boon. *International Journal of Pharmacognosy and Phytochemical Research*.

[18] Ministry of Health of the Republic of Indonesia (2020). *Indonesian Pharmacopoeia*.

[19] Tiwari P., Kumar B., Kaur M., Kaur G., Kaur H. (2011). Phytochemical screening and extraction. *Internationale Pharmaceutica Sciencia*.

[20] Beena P., Rajesh K. J., Arul B. (2016). Preliminary phytochemical screening of Cicer arietinum in folklore medicine for hepatoprotection. *Journal of Innovation in Pharmacy and Biological Science*.

[21] Awouters F., Niemegeers C. J. E., Lenaerts F. M., Janssen P. A. (2011). Delay of castor oil diarrhoea in rats: a new way to evaluate inhibitors of prostaglandin biosynthesis. *Journal of Pharmacy and Pharmacology*.

[22] Dassault Systèmes (2016). Discovery Studio 2016 Client®.

[23] Wibowo S., Widyarti S., Sabarudin A., Soeatmadji D. W., Sumitro S. T. (2019). The the role of astaxanthin compared with metformin in preventing glycated human serum albumin from possible unfolding: a molecular dynamic study. *Asian Journal of Pharmaceutical and Clinical Research*.

[24] Gautam G. (2018). Isolation and characterization of secondary metabolites from carica papaya leaves.

[25] Autodock The Scripps research institute: autodock tools 4.2.

[26] Kelutur F. J., Mustarichie R. (2020). Molecular docking of the potential compounds from cocoa shells (*Theobroma cacao* L.) against androgen receptor as anti-alopecia. *Journal of Global Pharma Technology*.

[27] Roy D., Kumar V., Acharya K. K., Thirumurugan K. (2014). Probing the binding of syzygium derived *α*-glucosidase inhibitors with N- and C-terminal human maltase glucoamylase by docking and molecular dynamics simulation. *Applied Biochemistry and Biotechnology*.

[28] Ordog R., Grolmusz V. (2008). Evaluating genetic algorithms in protein-ligand docking. *Bioinformatics Research and Applications*.

[29] Pratama M. R. F. (2016). Studi docking molekular senyawa turunan kuinolin terhadap reseptor estrogen alpha. *Jurnal Surya Medika*.

[30] Kim R., Skolnick J. (2008). Assessment of programs for ligand binding affinity prediction. *Journal of Computational Chemistry*.

[31] Kelutur F. J., Saptarini N. M., Mustarichie R., Kurnia D. (2022). Molecular docking of the terpenes in gorgonian corals to COX-2 and iNOS enzymes as anti-inflammatory. *Letters in Drug Design and Discovery*.

[32] Prabhu A. K., Devadas S. M., Lobo R., Udupa P., Chawla K., Ballal M. (2017). Antidiarrheal activity and phytochemical analysis of carica papaya fruit extract. *Journal of Pharmaceutical Sciences and Research*.

[33] Unaeze B. C., Ochiabuto O. M.-T. B., Ejike E. C., Obi M. C., Nwankpa S. N. (2018). Antimicrobial activities of Carica papaya leaf against diarrhoea causing agents. *International Journal of Advanced Engineering Research and Science*.

[34] Santana L. F., Inada A. C., Espirito Santo B. L. S. d. (2019). Nutraceutical potential of *Carica papaya* in metabolic syndrome. *Nutrients*.

[35] Sangsoy K., Mongkolporn O., Imsabai W., Luengwilai K. (2017). Papaya carotenoids increased in oxisols soils. *Agriculture and Natural Resources*.

[36] Zavala M. A., Perez S., Pérez C., Vargas R., Perez R. M. (1998). Antidiarrhoeal activity of *Walfheria americana, Commelina coelestis* and *Alternanthera repeus*. *Journal of Ethnopharmacology*.

[37] Izzo A. A., Mascolo N., Capasso R., Germano M. P., De Pasquale R., Capasso F. (1999). Inhibitory effect of cannabinoid agonists on gastric emptying in the rat. *Naunyn-Schmiedeberg’s Archives of Pharmacology*.

[38] Ammon H. V., Thomas P. J., Phillips S. F. (1974). Effects of oleic and ricinoleic acids on net jejunal water and electrolyte movement. Perfusion studies in man. *Journal of Clinical Investigation*.

[39] Terefe L., Nardos A., Debella A. (2023). Antidiarrheal activities of the methanol leaf extracts of olinia rochetiana (oliniaceae) against Castor oil-induced diarrhea in mice. *Journal of Experimental Pharmacology*.

[40] Hurwitz L., Weissinger J. (1980). Effects of variations in extracellular acetylcholine and calcium ion concentration on the operational level of calcium channels in intestinal smooth muscle. *Journal of Pharmacology and Experimental Therapeutics*.

[41] Adrian Md., Chy Md.N. U., Kamal A. T. M. M. (2019). Investigation of the biological activities and characterization of bioactive constituents of *Ophiorrhiza rugosa* var. prostrata (D.Don) & Mondal leaves through *in vivo*, *in vitro*, and *in silico* approaches. *Molecules*.

[42] Dubreuil J. (2013). Antibacterial and antidiarrheal activities of plant products against enterotoxinogenic *Escherichia coli*. *Toxins*.

[43] Okeke M. I., Iroegbu C. U., Eze E. N., Okoli A. S., Esimone C. O. (2001). Evaluation of extracts of the root of *Landolphia owerrience* for antibacterial activity. *Journal of Ethnopharmacology*.

[44] Aziz M. A., Mehedi M., Akter M. I., Sajon S. R., Mazumder K., Rana M. S. (2019). *In vivo* and *in silico* evaluation of analgesic activity of *Lippia alba*. *Clinical Phytoscience*.

[45] Carlo G. D., Mascolo N., Izzo A. A., Capasso F., Autore G. (1994). Effects of quercetin on the gastrointestinal tract in rats and mice. *Phytotherapy Research*.

[46] Daswani P. G., Brijesh S., Tatali P., Antia N. H., Birdi J. J. (2010). Antidiarrhoeal activity of *Zingiber officinale* (rosc.). *Current Science*.

[47] Verma N., Singh A., Gupta A., Sahu P., Rao C. (2011). Antidiarrheal potential of standardized extract of *Rhododendron arboretum*. *Indian Journal of Pharmacology*.

[48] Ekor M. (2014). The growing use of herbal medicines: issues relating to adverse reactions and challenges in monitoring safety. *Frontiers in Pharmacology*.

[49] Goyal M., Sasmal D., Nagori B. P. (2012). Review on ethnomedicinal uses, pharmacological activity and phytochemical constituents of *Ziziphus mauritiana* environment health perspective. *Medicine, Environmental Science, Agricultural and Food Sciences*.

[50] Kumar B., Divakar K., Tiwari P., Goli D. (2010). Evaluation of the anti-diarrhoeal effect of aqueous and ethanolic extracts of fruit pulp of *Terminalia belerica* in rats. *International Journal of Drug Development & Research*.

[51] Di Carlo G., Mascolo N., Izzo A. A., Capasso F. (1999). Flavonoids: old and new aspects of a class of natural therapeutic drugs. *Life Sciences*.

[52] Galvez J., Zarzuelo A., Crespo M. E., Lorente M. D., Ocete M. A., Jimenez J. (1993). Antidiarrhoeic activity of *Euphorbia hirta* extract and isolation of an active flavonoid constituent. *Planta Medica*.

[53] Haque T., Shams R., Tahsin F. (2019). Antidiarrheal activity of methanol extract of *Piper sylvaticum* (Roxb.) stems in mice and *in silico* molecular docking of its isolated compounds. *Discovery Phytomedicine*.

[54] Pangastuti A., Amjn M., Indriwati E. S. (2016). Discovering the potential of natural compound based on reverse docking. *Proceedings of the 2016 National Seminar II, Cooperation of the Biology Education Study Program with the Center for Population and Environment Studies Universitas Muhammadiya Malang*.

[55] Adelina R. (2014). Molecular docking studies of annomuricin E and muricapentocin on antiproliferation activity. *Indonesian Journal of Pharmaceutical Sciences*.

[56] Umamaheswari M., Madeswaran A., Asokkumar K. (2013). Virtual screening analysis and *in vitro* xanthine oxidase inhibitory activity of some commercially available flavonoids. *Iranian Journal of Pharmaceutical Research: International Journal of Physics and Research*.

[57] Kartasasmita R. E., Herowati R., Harmastuti N., Gusdinar T. (2010). Quercetin derivatives docking based on study of flavonoids interaction to cyclooxygenase-2. *Indonesian Journal of Chemistry*.

[58] O’Hagan S., Kell D. B. (2015). The apparent permeabilities of Caco-2 cells to marketed drugs: magnitude, and independence from both biophysical properties and endogenite similarities. *PeerJ*.

[59] Megantara S., Levita J., Iwo M. I., M I., Ibrahim S. (2018). Absorption, distribution, and toxicity prediction of andrograpolide and its derivatives as anti-HIV drugs. *Research Journal of Chemistry and Environment*.

[60] Han Y., Zhang J., Hu C. Q., Zhang X., Ma B., Zhang P. (2019). *In silico* ADME and toxicity prediction of ceftazidime and its impurities. *Frontiers in Pharmacology*.

[61] Muttaqin F. Z., Pratama M. F., Kurniawan F. (2019). Molecular docking and molecular dynamic studies of stilbene derivative compounds as Sirtuin-3 (SIRT3) histone deacetylase inhibitor on melanoma skin cancer and their toxicities. *Journal of Pharmacopolium*.

[62] Feltrin C., Farias I. V., Sandjo L. P., Reginatto F. H., Simoes C. M. O. (2020). Effects of standardized medicinal plant extracts on drug metabolism mediated by CYP3A4 and CYP2D6 enzymes. *Chemical Research in Toxicology*.

[63] Yao W. R., Wang H. Y., Wang S. T., Sun S. L., Zhou J., Luan Y. Y. (2011). Assessment of the antibacterial activity and the antidiarrheal function of flavonoids from bayberry fruit. *Journal of Agricultural and Food Chemistry*.

[64] Nugroho A., Heryani H., Choi J. S., Park H.-J. (2017). Identification and quantification of flavonoids in *Carica papaya* leaf and peroxynitrite-scavenging activity. *Asian Pacific Journal of Tropical Biomedicine*.

[65] Sharma A., Bachheti A., Sharma P., Bachheti R. K., Husen A. (2020). Phytochemistry, pharmacological activities, nano-particle fabrication, commercial products and waste utilization of Carica papaya L.: a comprehensive review. *Current Research in Biotechnology*.

[66] Aslam M., Tan C. K., Prayitno A. (2003). *Clinical Pharmacy: Towards Rational Medicine and Respect for Patient Choice*.

