# Supplementary material for: Exploring the Antidiarrheal Properties of Papaya Leaf: Insights In Vivo Study in Mice-Model and In Silico Analysis at M3 Muscarinic Acetylcholine Receptor Interaction
Source: Scientifica (Cairo). 2024 Jun 12;2024:1558620. doi: 10.1155/2024/1558620 (PMC11221971; doi:10.1155/2024/1558620)
Supplement: Supplementary Materials — Docking results of triplicates of phytochemical constituents of papaya leaves to M3-AchR. [file 1558620.f1.docx]

**Docking results of triplicates of phytochemical constituents of papaya leaves to M_3_-AchR**

| **Compounds** | **Amino acid residue interaction** | | **ΔG (Kcal/mol)** | | | | | | | | **Ki (nM)** | | | | | |
| --- | --- | --- | --- | --- | --- | --- | --- | --- | --- | --- | --- | --- | --- | --- | --- | --- |
|  | **Hydrogen bond** | **Van der Waals bond** |  | | | | | | | |  | | | | | |
| **Alkaloids** | | | | | | | | | | | | | |  |  | |
| Carpaine | Ser151 | Phe221, Ile222, Pro228, Thr234, Trp199, Ala235, Ala238, Phe239, Asn507, Val155, Leu225, Tyr148 | +22.74 | | | | +22.74 | | | +22.75 | - | | | - | - | |
| Dehydrocarpaine I | - | Ser151, Trp503, Val155, Asn152, Thr234, Ala235, Val510, Thr231, Trp525, Tyr529 | -5.34 | | | | -5.34 | | | -5.34 | 122660 | | | 122810 | 122770 | |
| Dehydrocarpaine II | Ser151 | Trp525, Ile222, Val510, Thr234, Ala235, Trp199, Ala238, Asn152, Val155 | +10.67 | | | | +10.67 | | | +10.67 | - | | | - | - | |
| Emetine | Ser151, Asn507, Asn152, Ala238, Trp503, Asp147 | Tyr533, Ile116, Tyr529, Thr231, Val510, Thr234, Val155 | -7.18 | | | -7.25 | | | | -7.15 | 5500 | | | 4830 | 5760 | |
| **Flavonoids and flavonoid glycosides** | | | | | | | | | | | | | | | | |
| Quercetin 3-(2-rhamnosylrutinoside) | Ala235, Phe239, Trp199, Asn152, Ser151, Tyr148, Tyr529, Tyr506 | Ile116, Phe221, Ile222, Thr231, Val510, Thr234, Val155, Asn507 | -7.98 | -8.50 | | | | | | -7.95 | 1420 | | | 588.59 | 1490 | |
| Kaempferol 3-(2-rhamnosylrutinoside) | Thr231, Ala235, Phe239, Asn152, Trp199, Ser151, Tyr506, Tyr148, Tyr529 | Ile116, Tyr533, Ile222, Phe221, Leu225, Thr234, Asn507, Val155 | -7.91 | -7.76 | | | | | | -7.90 | 1600 | | | 2050 | 1610 | |
| Quercetin 3-rutinoside | Tyr529, Tyr506, Leu225 | Asn152, Ser151, Trp503, Ile116, Tyr533, Asn507, Phe239, Ala235, Thr231, Thr234, Ser226, Phe221, Ile222 | -11.50 | -11.49 | | | | | | -11.50 | 3.74 | | | 3.78 | 3.72 | |
| Myricetin 3-rhamnoside | Ser151, Tyr529, Ala235 | Gly528, Asn507, Phe239, Thr234, Thr231, Asn152, Val155, Trp503, Ile116, Tyr533 | -10.74 | -10.73 | | | | | | -10.74 | 13.51 | | | 13.53 | 13.52 | |
| Quercetin | Trp199, Asn152, Ser151, Tyr529, Cys532, Tyr533 | Ile116, Ala235, Phe239, Tyr506 | -8.66 | -8.66 | | | | | | -8.66 | 448.44 | | | 450.74 | 451.02 | |
| Kaempferol | Asn152, Trp199, Ser151, Tyr529, Tyr533, Cys532 | Ala235, Phe239, Tyr506 | -8.57 | -8.57 | | | | | | -8.58 | 523.82 | | | 520.28 | 515.56 | |
| **Phenol and phenolic acid** | | | | | | | | | | | | | | | | |
| Chlorogenic acid | Ser151, Tyr529, Tyr506, Tyr533 | Asn152, Ala238, Thr234, Trp199, Ala235, Phe239, Trp503, Asn507, Asp147 | -8.49 | | -8.53 | | | | -8.58 | | 549.26 | | 559.35 | | 512.17 | |
| Ferulic acid | Asn507, Cys532, Trp503, Asn152, Trp199 | Phe239, Ala238, Ser151, Tyr148, Tyr529 | -5.49 | | -5.51 | | | | -5.49 | | 94560 | | 92030 | | 94870 | |
| Gallic acid | Tyr148, Trp199, Asn152, Ser151 | Val155, Phe239, Trp503, Tyr529 | -4.62 | | -4.62 | | | | -4.62 | | 412330 | | 410880 | | 412410 | |
| 5,7-Dimethoxycoumarin | Asn152, Cys532, Asp147 | Trp199, Tyr148, Ser151, Tyr529, Tyr506, Asn507, Trp503 | -6.56 | | -6.56 | | | | -6.56 | | 15550 | | 15540 | | 15540 | |
| Caffeic acid | Cys532, Asn507, Asn152, Trp199 | Phe239, Ser151, Trp503, Ala238 | -5.58 | | -5.58 | | | | -5.58 | | 81800 | | 81460 | | 81070 | |
| o-Coumaric acid | Ser151, Asn507, Tyr148 | Asp147, Trp503, Phe239, Tyr506 | -5.75 | | -5.75 | | | | -5.75 | | 61340 | | 61430 | | 61440 | |
| p-Coumaric acid | Cys532, Asn507, Trp199, Asn152 | Tyr148, Phe239, Ala238 | -5.20 | | -5.20 | | | | -5.19 | | 154720 | | 154870 | | 155680 | |
| Protocatechuic acid | Trp199, Asn152, Tyr148, Ser151 | Trp503, Val155, Tyr506, Tyr529 | -4.55 | | -4.56 | | | | -4.56 | | 464150 | | 451060 | | 451140 | |
| (*E*)-3-(hydroxy-3-(3,4,5-trimethoxybenzyl) phenyl) acrylic acid | Ile222, Ser151, Tyr148, Tyr529, Asp147 | Phe221, Cys532, Thr234, Trp199, Ala238, Asn152 | -8.29 | | -8.31 | | | | -8.29 | | 840.30 | | 806.02 | | 838.05 | |
| **Comparison (drug)** | | | | | | | | | | | | | | | | |
| Loperamide | Tyr506, Thr231, Ser151, Ile222, Thr231, Trp525, Tyr148, Cys532 | Tyr533, Phe239, Asn507, Ala235, Thr234, Trp199, Ala238, Asn152 | -11.69 | | -11.60 | | | -11.54 | | | 2.70 | 3.16 | | | | 3.45 |
